# Two-year persistence and compliance with osteoporosis therapies among postmenopausal women in a commercially insured population in the United States

**DOI:** 10.1007/s11657-017-0316-5

**Published:** 2017-02-28

**Authors:** Emily Durden, Lionel Pinto, Lorena Lopez-Gonzalez, Paul Juneau, Richard Barron

**Affiliations:** 1 Life Sciences, Truven Health Analytics, 7700 Old Georgetown Road, Bethesda, MD 20814 USA; 2 Global Health Economics, Amgen Inc., 1 Amgen Center Drive, Thousand Oaks, CA 91320 USA; 3 Custom Data Analytics, Life Sciences, Truven Health Analytics, 7700 Old Georgetown Road, Bethesda, MD 20814 USA; 4 Statistical Services Group, Truven Health Analytics, 7700 Old Georgetown Road, Bethesda, MD 20814 USA; 5 Global Health Economics, Amgen Inc., 1 Amgen Center Drive, Thousand Oaks, CA 91320 USA

**Keywords:** Persistence, Compliance, Osteoporosis, Bisphosphonates

## Abstract

***Summary*:**

This retrospective, observational study assessed 2-year persistence and compliance by treatment, route of administration, and dosing frequency in postmenopausal women initiating a new osteoporosis therapy. Two-year persistence and compliance rates were higher in women receiving injectables compared with oral agents.

**Purpose:**

This study extends previous studies limited to 1-year follow-up by examining persistence with osteoporosis therapies over a 2-year period and compares short- and long-term trends in persistence and compliance among postmenopausal women with commercial or Medicare supplemental insurance in the USA.

**Methods:**

This retrospective, observational cohort study enrolled women ≥50 years newly initiating osteoporosis therapy between January 1 and December 31, 2012 (i.e., the index date), with continuous enrollment ≥14 months before and ≥24 months after their index date. Persistence (continuous therapy without a >60-day gap) and compliance with the index therapy were evaluated at 2 years of follow-up. Multivariable logistic regression was used to compare the odds of persistence and compliance across treatment and dosing regimens.

**Results:**

This study included 43,543 patients with mean (standard deviation) age 65 (10) years. At 2 years of follow-up, persistence and compliance were higher for patients treated with injectable agents (ranging from 34 to 41%, excluding an every-3-month injection) than those treated with oral agents (ranging from 20 to 31%). Additionally, patients initiating oral bisphosphonates (except risedronate once daily), raloxifene (daily), or zoledronic acid (annually) had significantly lower odds of persistence compared with denosumab (every 6 months).

**Conclusions:**

Patients initiating injectable therapies had greater persistence and compliance at 2 years than those initiating oral therapies. Patients initiating an every-6-month injection had significantly higher persistence compared with those initiating more frequently dosed (e.g., daily and weekly) oral or injectable agents.

## Introduction

Osteoporosis is a chronic, systemic bone disorder that is characterized by low bone density, deterioration of bone tissue, loss of bone strength, and an increase in bone fracture risk [1]. Treatment goals for osteoporosis encompass managing bone fracture risk by minimizing bone loss, increasing bone mineral density (BMD), and improving bone microarchitecture [2, 3]. This is accomplished primarily through therapy with anti-resorptive agents, including bisphosphonates, estrogen receptor agonists and antagonists, parathyroid hormone or its analogues, and monoclonal antibody therapy [4].

Persistence and compliance are important for improving outcomes in patients with osteoporosis. Recent studies have shown that patients who are persistent and compliant have fewer total, vertebral, non-vertebral, and hip fractures [5, 6]. As persistence tends to decline over time, potentially leading to poorer outcomes, a long-term view of persistence is important in strategizing how to improve outcomes.

However, the majority of research to date has focused on persistence and compliance over a 1-year period, with limited information on long-term trends [7–11]. In these short-term studies, rates of persistence and compliance with oral bisphosphonates were generally poor (<50%) [12–17]. These studies have generally found that patients are more persistent and compliant with injectable osteoporosis therapies—including denosumab, teriparatide, and zoledronic acid—compared with oral osteoporosis therapies—including oral bisphosphonates [7, 8, 18–20]. Similarly, Cheng et al. recently found that the rate of persistence among patients receiving denosumab was 68%, compared with 29–35% for bisphosphonates, 42% for raloxifene, and 59% for teriparatide during 1 year of follow-up [7].

The primary objective of this study was to evaluate short- and long-term (i.e., 1- and 2-year) persistence and compliance with osteoporosis therapies among postmenopausal women with osteoporosis with commercial or Medicare supplemental insurance. In addition, this study compared short- and long-term persistence and compliance across different osteoporosis therapies and dosing regimens.

## Methods

### Study design

This was a retrospective, observational cohort study using administrative claims data from the *MarketScan*® databases, including the Commercial Claims and Encounters (Commercial), Medicare Supplemental and Coordination of Benefits (Medicare), and Early View databases. Patients were followed for at least 2 years after their index date—the date of their first new dispensing or medical claim for one of the osteoporosis therapies of interest (i.e., the index therapy), between January 1, 2012, and December 31, 2014. Persistence and compliance outcomes were measured at 12 and 24 months of follow-up.

### Patients

Patients were included in this study if they were female; aged 50 years or older on their index date; had at least one pharmacy or medical claim for a Food and Drug Administration—approved osteoporosis therapy any time between January 1, 2012, and December 31, 2012; had at least 14 months of continuous enrollment with medical and pharmacy benefits before the index date (pre-index period); and had at least 24 months of continuous enrollment with medical and pharmacy benefits after the index date (post-index period).

Patients were excluded from this study if they had a diagnosis or evidence of the following during the pre-index period: treatment with the index therapy, Paget disease of the bone or other osteitis deformans and osteopathies, osteogenesis imperfecta, hypercalcemia, malignant cancer, human immunodeficiency virus infection, or preventive treatment for risk of breast cancer. Patients were also excluded if they had a cancer or metastasis diagnosis before a medical claim for denosumab or zoledronic acid in the post-index period (for patients receiving denosumab and zoledronic acid only), as these therapies may have been used in cancer rather than osteoporosis in these patients.

Treatment groups included injectable and oral therapies, as well as a range of dosing frequencies. The injectable therapies were denosumab subcutaneous (SC) injection every 6 months (Q6M), ibandronate intravenous (IV) injection every 3 months (Q3M), teriparatide daily SC, and zoledronic acid annual IV. The oral therapies were alendronate daily, alendronate weekly, ibandronate monthly, raloxifene daily, risedronate daily, risedronate weekly, and risedronate monthly.

### Outcomes

Persistence was measured at 12 and 24 months after the index date and was defined as remaining on the index therapy during the respective follow-up period with no gap of >60 days following the fill date plus the days’ supply of the previous claim. Compliance was measured at 12 and 24 months after the index date and was defined using the medication possession ratio (MPR), or the total days supplied from all claims of the index therapy divided by the length of follow-up. Patients were considered compliant if the MPR was ≥0.80. Sensitivity analyses were also conducted for persistence outcomes using allowed gap periods of 30 or 90 days, instead of 60 days, over the course of 24 months.

### Statistical analysis

Baseline patient characteristics and persistence and compliance outcomes were summarized using descriptive statistics. Multivariable logistic regression models were estimated to compare persistence and compliance across treatment/dosing regimens. Odds ratios (ORs) were calculated from logistic regression models comparing persistence and compliance with the Q6M injectable therapy (denosumab) with the other osteoporosis therapies of interest, adjusting for demographic and clinical characteristics at baseline (Table 3). The models also incorporated adjusted propensity score weights.

## Results

### Patient disposition

**Fig. 1:**
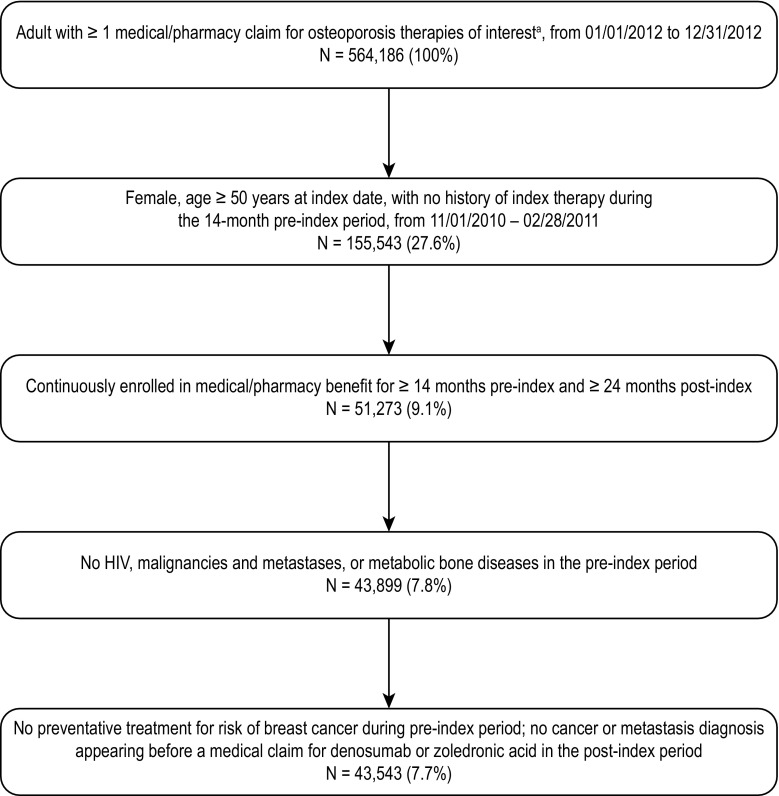
Patient selection. ^a^Denosumab SC Q6M, alendronate QD oral, alendronate QW oral, ibandronate QM oral, ibandronate IV Q3M, risedronate QD oral, risedronate QW oral, risedronate QM oral, raloxifene, teriparatide SC QD, and zoledronic acid IV annual. *HIV* human immunodeficiency virus, *IV* intravenous, *QD* once daily, *QM* once monthly, *Q3M* once every 3 months, *Q6M* once every 6 months, *QW* once a week, *SC* subcutaneous

A total of 564,186 adult patients were identified who had at least one pharmacy or medical claim for an osteoporosis therapy of interest between January 1, 2012, and December 31, 2012 (Fig. 1). Of those, a total of 43,543 patients met the eligibility criteria: 3599 (8.3%) received denosumab Q6M SC, 224 (0.5%) received alendronate daily, 19,486 (44.8%) received alendronate weekly, 5981 (13.7%) received ibandronate monthly, 165 (0.4%) received ibandronate IV Q3M, 3423 (7.9%) received raloxifene daily, 53 (0.1%) received risedronate daily, 2968 (6.8%) received risedronate weekly, 1986 (4.6%) received risedronate monthly, 1100 (2.5%) received teriparatide SC daily, and 4558 (10.5%) received zoledronic acid IV annually.

### Demographic and clinical characteristics at baseline

Overall, patients had a mean (standard deviation) age of 65 (10) years, 57.9% were below the age of 65 years, 51.4% were enrolled in a preferred provider organization health plan, and 84.1% were urban residents (Table 1). During the baseline study period, 26.4 and 12.2% of patients had a diagnosis of osteoporosis and osteopenia, respectively. Among the study population, 7.8% of patients had experienced an osteoporosis-related fracture, while 36.7% were at a high risk for fracture, as defined by age 70 years or older or having had an osteoporotic fracture during the baseline period (Table 2).

**Table 1:** Demographic and clinical characteristics at baseline

Characteristic	All *N* = 43,543	Denosumab SC Q6M *n* = 3599	Alendronate QD *n* = 224	Alendronate QW *n* = 19,486	Ibandronate QM *n* = 5981	Ibandronate IV Q3M *n* = 165	Raloxifene QD *n* = 3423	Risedronate QD *n* = 53	Risedronate QW *n* = 2968	Risedronate QM *n* = 1986	Teriparatide SC QD *n* = 1100	Zoledronic acid IV annually *n* = 4558
Age, mean (SD)	64.9 (10.4)	68.4 (11.1)	65.0 (10.5)	64.9 (10.6)	63.1 (9.8)	68.2 (10.9)	62.6 (9.1)	68.2 (12.3)	63.8 (10.1)	63.2 (9.7)	67.2 (10.8)	66.7 (10.4)
Age groups, *n* (%)												
45–64 years	25,212 (57.9)	1577 (43.8)	126 (56.3)	11,221 (57.6)	3903 (65.3)	74 (44.8)	2331 (68.1)	23 (43.4)	1884 (63.5)	1324 (66.7)	532 (48.4)	2217 (48.6)
≥65 years	18,331 (42.1)	2022 (56.2)	98 (43.8)	8265 (42.4)	2078 (34.7)	91 (55.2)	1092 (31.9)	30 (56.6)	1084 (36.5)	662 (33.3)	568 (51.6)	2341 (51.4)
Health insurance type, *n* (%)												
Comprehensive	10,525 (24.2)	1033 (28.7)	58 (25.9)	5008 (25.7)	1377 (23.0)	55 (33.3)	684 (20.0)	21 (39.6)	472 (15.9)	260 (13.1)	304 (27.6)	1253 (27.5)
POS	3495 (8.0)	258 (7.2)	21 (9.4)	1530 (7.9)	528 (8.8)	16 (9.7)	314 (9.2)	3 (5.7)	268 (9.0)	189 (9.5)	88 (8.0)	280 (6.1)
HMO	4155 (9.5)	262 (7.3)	31 (13.8)	2143 (11.0)	463 (7.7)	7 (4.2)	244 (7.1)	4 (7.5)	263 (8.9)	180 (9.1)	75 (6.8)	483 (10.6)
PPO	22,366 (51.4)	1866 (51.8)	99 (44.2)	9333 (47.9)	3206 (53.6)	81 (49.1)	1918 (56.0)	19 (35.8)	1719 (57.9)	1229 (61.9)	563 (51.2)	2333 (51.2)
Other	2432 (5.6)	135 (3.8)	15 (6.7)	1217 (6.2)	338 (5.7)	5 (3.0)	217 (6.3)	6 (11.3)	177 (6.0)	109 (5.5)	60 (5.5)	153 (3.4)
Unknown	570 (1.3)	45 (1.3)	0 (0.0)	255 (1.3)	69 (1.2)	1 (0.6)	46 (1.3)	0 (0.0)	69 (2.3)	19 (1.0)	10 (0.9)	56 (1.2)
Geographic region, *n* (%)												
Northeast	8291 (19.0)	643 (17.9)	35 (15.6)	3292 (16.9)	1130 (18.9)	54 (32.7)	748 (21.9)	8 (15.1)	781 (26.3)	638 (32.1)	215 (19.5)	747 (16.4)
North Central	12,419 (28.5)	937 (26.0)	71 (31.7)	6172 (31.7)	1410 (23.6)	38 (23.0)	922 (26.9)	19 (35.8)	620 (20.9)	335 (16.9)	312 (28.4)	1583 (34.7)
South	17,036 (39.1)	1589 (44.2)	82 (36.6)	7111 (36.5)	2663 (44.5)	46 (27.9)	1317 (38.5)	19 (35.8)	1187 (40.0)	793 (39.9)	440 (40.0)	1789 (39.2)
West	5431 (12.5)	406 (11.3)	36 (16.1)	2760 (14.2)	721 (12.1)	27 (16.4)	391 (11.4)	7 (13.2)	349 (11.8)	193 (9.7)	127 (11.5)	414 (9.1)
Unknown	366 (0.8)	24 (0.7)	0 (0.0)	151 (0.8)	57 (1.0)	0 (0.0)	45 (1.3)	0 (0.0)	31 (1.0)	27 (1.4)	6 (0.5)	25 (0.5)
Urban resident, *n* (%)	36,614 (84.1)	3016 (83.8)	193 (86.2)	16,263 (83.5)	5100 (85.3)	147 (89.1)	2864 (83.7)	42 (79.2)	2649 (89.3)	1748 (88.0)	911 (82.8)	3681 (80.8)

*HMO* health maintenance organization, *IV* intravenous, *POS* point of service, *PPO* preferred provider organization, *QD* once daily, *QM* once monthly, *Q3M* once every 3 months, *Q6M* once every 6 months, *QW* once a week, *SC* subcutaneous, *SD* standard deviation

**Table 2:** Clinical characteristics during baseline

Characteristic	All *N* = 43,543	Denosumab SC Q6M *n* = 3599	Alendronate QD *n* = 224	Alendronate QW *n* = 19,486	Ibandronate QM *n* = 5981	Ibandronate IV Q3M *n* = 165	Raloxifene QD *n* = 3423	Risedronate QD *n* = 53	Risedronate QW *n* = 2968	Risedronate QM *n* = 1986	Teriparatide SC QD *n* = 1100	Zoledronic acid IV annually *n* = 4558
CCI, mean (SD)	0.6 (1.0)	0.8 (1.2)	0.6 (0.9)	0.6 (1.0)	0.5 (0.9)	0.9 (1.2)	0.4 (0.8)	0.6 (1.0)	0.5 (1.0)	0.5 (0.9)	0.8 (1.2)	0.7 (1.1)
Unique ICD-9-CM diagnoses, mean (SD)	12 (7)	14 (8)	11 (7)	11 (7)	11 (7)	15 (9)	11 (7)	13 (7)	12 (7)	12 (7)	16 (9)	14 (8)
Unique osteoporosis drugs received, mean (SD)	12 (9)	14 (10)	12 (8)	11 (9)	11 (9)	15 (11)	10 (8)	14 (11)	11 (9)	11 (8)	16 (12)	14 (10)
Diagnosis of osteoporosis, *n* (%)	11,508 (26.4)	2053 (57.0)	27 (12.1)	3274 (16.8)	1137 (19.0)	82 (49.7)	714 (20.9)	12 (22.6)	718 (24.2)	483 (24.3)	764 (69.5)	2244 (49.2)
Diagnosis of osteopenia, *n* (%)	5291 (12.2)	337 (9.4)	27 (12.1)	2154 (11.1)	794 (13.3)	20 (12.1)	576 (16.8)	3 (5.7)	438 (14.8)	288 (14.5)	132 (12.0)	522 (11.5)
Osteoporosis therapy, *n* (%)	5721 (13.1)	1007 (28.0)	18 (8.0)	1887 (9.7)	773 (12.9)	38 (23.0)	423 (12.4)	5 (9.4)	444 (15.0)	227 (11.4)	277 (25.2)	622 (13.6)
Osteoporosis-related fractures, *n* (%)	3388 (7.8)	394 (10.9)	21 (9.4)	1441 (7.4)	340 (5.7)	9 (5.5)	158 (4.6)	6 (11.3)	190 (6.4)	120 (6.0)	334 (30.4)	375 (8.2)
High risk for fracture, *n* (%)	15,981 (36.7)	1825 (50.7)	79 (35.3)	7191 (36.9)	1741 (29.1)	75 (45.5)	891 (26.0)	26 (49.1)	937 (31.6)	571 (28.8)	614 (55.8)	2031 (44.6)
Renal insufficiency, *n* (%)	1217 (2.8)	196 (5.4)	5 (2.2)	568 (2.9)	134 (2.2)	4 (2.4)	53 (1.5)	1 (1.9)	61 (2.1)	50 (2.5)	46 (4.2)	99 (2.2)
GI side effects, *n* (%)	6230 (14.3)	690 (19.2)	34 (15.2)	2235 (11.5)	764 (12.8)	44 (26.7)	517 (15.1)	5 (9.4)	436 (14.7)	260 (13.1)	209 (19.0)	1036 (22.7)

*CCI* Charlson Comorbidity Index, *GI* gastrointestinal, *ICD-9-CM* International Classification of Diseases, Ninth Revision, Clinical Modification, *IV* intravenous, *QD* once daily, *QM* once monthly, *Q3M* once every 3 months, *Q6M* once every 6 months, *QW* once a week, *SC* subcutaneous, *SD* standard deviation

### Twelve-month persistence and compliance

**Fig. 2:**
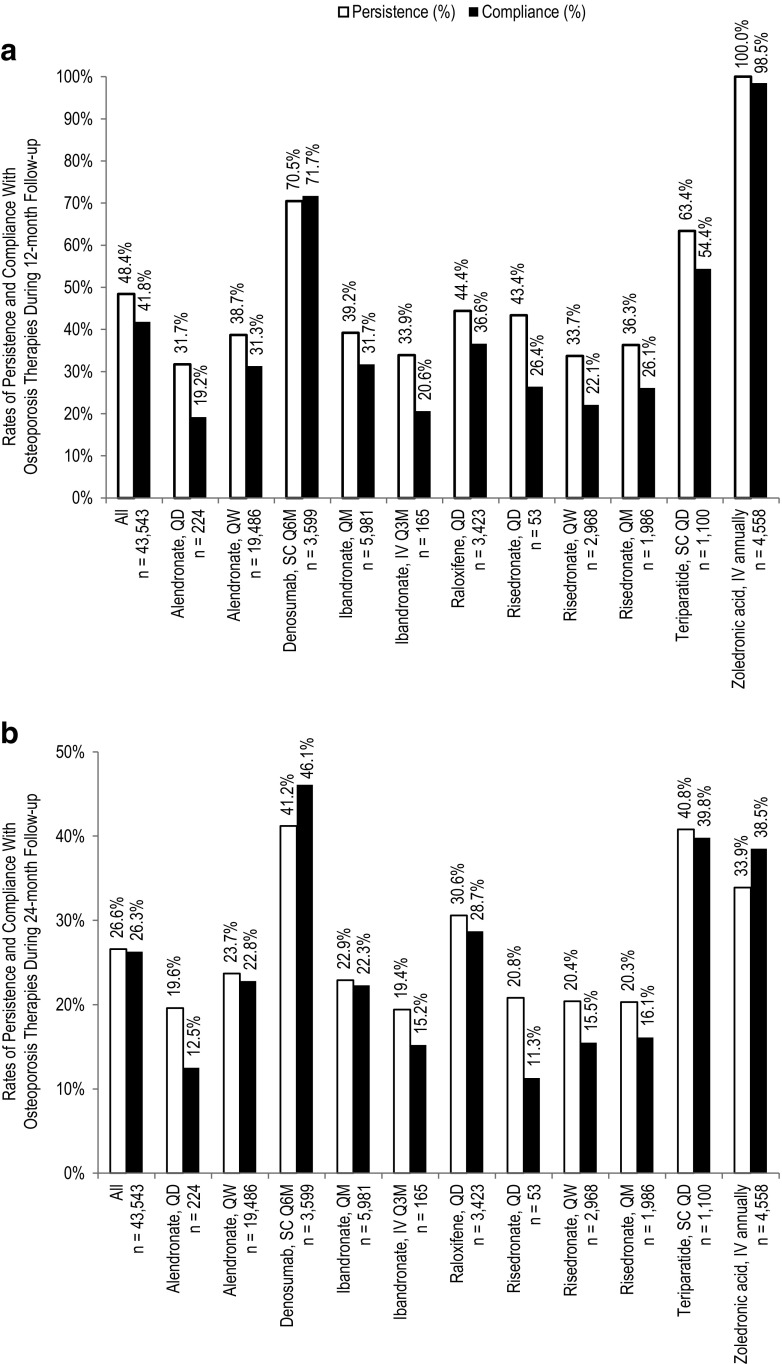
Rates of persistence and compliance with osteoporosis therapies during 12- and 24-month follow-up. Rates of persistence (*white bars*) and compliance (*black bars*) among women receiving osteoporosis therapies of interest during **a** 12-month and **b** 24-month follow-up. *IV* intravenous, *QD* once daily, *QM* once monthly, *Q3M* once every 3 months, *Q6M* once every 6 months, *QW* once a week, *SC* subcutaneous

Overall, 48.4 and 41.8% of patients were persistent and compliant at 12 months of follow-up, respectively (Fig. 2a). In general, patients receiving injectable therapies had higher rates of persistence and compliance (83.4 and 81.7%, respectively) compared with those receiving oral therapies (38.8 and 30.7%, respectively). Among patients receiving injectable therapies, those receiving a therapy with a Q6M dosing frequency (i.e., denosumab) had higher rates of persistence and compliance (70.5 and 71.7%, respectively) than those receiving a therapy with a daily dosing frequency (i.e., teriparatide): 63.4 and 54.4%, respectively; the sample size for the Q3M dosing (i.e., ibandronate) was too small for meaningful analyses. Rates of persistence with oral therapies ranged from 31.7 to 44.4% among individual therapies (*P* < 0.001 across all groups). Rates of compliance with oral therapies ranged from 19.2 to 36.6% for individual therapies (*P* < 0.001 across all groups).

### Twenty-four-month persistence and compliance

Rates of persistence and compliance were lower at 24 than 12 months. Overall, 26.6 and 26.3% of patients were persistent and compliant at 24 months of follow-up, respectively (Fig. 2b). Among patients receiving injectable therapies, rates of persistence and compliance were 37.2 and 41.1%, respectively. Again, rates of persistence and compliance were higher among patients receiving injectable therapy with Q6M dosing (41.2 and 46.1%, respectively) compared with daily dosing (40.8 and 39.8%, respectively). Among patients receiving oral therapies, overall rates of persistence were 23.7, ranging from 19.6 to 30.6% among individual therapies, while overall rates of compliance were 22.2%, ranging from 11.3 to 28.7% among individual therapies (*P* < 0.001 across all groups).

### Adjusted odds of persistence and compliance

Multivariable logistic regression models were estimated to compare the odds of persistence and compliance with denosumab SC and other osteoporosis therapies of interest. At 12 months, patients initiating other osteoporosis therapies were significantly less likely to be persistent (OR ranging from 0.07 to 0.25, *P* < 0.001 for all) and compliant (OR ranging from 0.09 to 0.46, *P* < 0.001 for all) compared with those initiating denosumab SC (Table 3).

**Table 3:** Propensity score weight-adjusted odds ratios from logistic regression models, persistence and compliance (60-day gap allowance) with index therapy over 12 and 24 months

Denosumab vs therapy	12-month follow-up, OR (95% CI)		24-month follow-up, OR (95% CI)	
	Persistence	Compliance	Persistence	Compliance
Alendronate QD	0.07 (0.05, 0.11)	0.09 (0.06, 0.15)	0.36 (0.22, 0.59)	0.17 (0.10, 0.30)
Alendronate QW	0.10 (0.09, 0.11)	0.18 (0.16, 0.20)	0.45 (0.40, 0.51)	0.35 (0.31, 0.39)
Ibandronate QM	0.10 (0.09, 0.11)	0.18 (0.16, 0.21)	0.44 (0.38, 0.50)	0.34 (0.30, 0.39)
Ibandronate IV Q3M	0.08 (0.05, 0.12)	0.10 (0.06, 0.17)	0.34 (0.20, 0.60)	0.21 (0.11, 0.38)
Raloxifene QD	0.12 (0.10, 0.14)	0.22 (0.19, 0.26)	0.64 (0.55, 0.74)	0.47 (0.41, 0.55)
Risedronate QD	0.12 (0.06, 0.26)	0.15 (0.06, 0.35)	0.39 (0.15, 1.03)*	0.15 (0.05, 0.52)
Risedronate QW	0.08 (0.07, 0.09)	0.11 (0.09, 0.13)	0.37 (0.32, 0.44)	0.22 (0.18, 0.26)
Risedronate QM	0.09 (0.07, 0.10)	0.14 (0.12, 0.17)	0.37 (0.31, 0.44)	0.23 (0.19, 0.28)
Teriparatide SC QD	0.25 (0.20, 0.30)	0.46 (0.38, 0.57)	0.95 (0.77, 1.16)†	0.76 (0.62, 0.93)
Zoledronic acid IV annually	n/a	n/a	0.76 (0.67, 0.86)	0.76 (0.67, 0.86)

Models adjusted for age, health plan type, US Census region, urban residency, patient out-of-pocket expenditure for the index therapy, and the following baseline clinical characteristics: Charlson Comorbidity Index score, number of unique medications prescribed, diagnosis of osteoporosis, osteopenia, renal insufficiency, gastrointestinal disorders, coronary heart disease, and osteoporosis-related fracture. *CI* confidence interval, *IV* intravenous, *n/a* not applicable, *OR* odds ratio, *QD* once daily, *QM* once monthly, *Q3M* once every 3 months, *QW* once a week, *SC* subcutaneous. *P* < 0.0001 for denosumab compared with all osteoporosis therapies

*P = 0.063; †*P* = 0.997

At 24 months, patients initiating other osteoporosis therapies were significantly less likely to be persistent compared with those initiating denosumab SC (OR ranging from 0.36 to 0.76, *P* < 0.001), with the exception of risedronate daily and teriparatide SC (OR 0.39 and 0.95, respectively; *P* = 0.063 and *P* = 0.997, respectively; Table 3). Patients initiating other osteoporosis therapies were significantly less likely to be compliant compared with those initiating denosumab SC (OR ranging from 0.15 to 0.76, *P* < 0.001 for all; Table 3).

### Sensitivity analyses

ORs were also calculated using allowable gaps in the index therapy of 30 and 90 days, instead of 60 days, over the course of 24 months. Results from these analyses using the alternative allowable gap periods did not differ substantively from those obtained using a 60-day gap allowance in the definition of persistence.

## Discussion

In this US-based analysis of women initiating a new osteoporosis therapy, persistence and compliance were higher among those treated with injectable therapies compared with oral osteoporosis therapies over both a 1- and 2-year follow-up period, with the exception of ibandronate IV, which had a very low sample size. Persistence and compliance over 24 months were higher among patients initiating denosumab SC, an injectable therapy administered Q6M, compared with those initiating other, more frequently dosed osteoporosis therapies, including oral or injectable bisphosphonates and raloxifene.

Previous studies of persistence and compliance with osteoporosis therapies are generally limited to 1 year of follow-up. Even within that time period, persistence and compliance with osteoporosis therapies are generally poor for oral osteoporosis therapies, while injectable osteoporosis therapies are generally associated with higher rates of both persistence and compliance. The 12-month results from this study are consistent with those of other published studies. One study that pooled the data of all osteoporosis therapies found rates of overall persistence and compliance to be 50 and 68%, respectively [21]. Rates of persistence with denosumab SC over 1 year have ranged between 63 and 82%, while rates of compliance have ranged between 71 and 91% [7, 19, 22]. Rates of persistence and compliance with teriparatide have been shown to be 54 and 32%, respectively, at 1 year of follow-up and 19 and 48%, respectively, at 2 years of follow-up [17, 20]. Because zoledronic acid is taken once a year, studies of persistence and compliance at 1 year yield predictably high rates. For oral bisphosphonates (daily, weekly, and monthly), rates of persistence ranged from 15 to 50%, while rates of compliance ranged from 39 to 43% [6, 12–16]. Rates of persistence with raloxifene ranged from 14 to 34% [12, 14, 15].

As persistence tends to decline over time, potentially leading to poorer outcomes, knowledge of a long-term view of persistence is important for developing strategies to improve persistence and, consequently, outcomes [23–25]. Poor persistence and compliance increase the risk of fractures [16, 23, 26, 27]. In a study of over 35,000 women aged 45 years and older from an administrative claims database, compliant patients had a 21% reduction in overall fracture risk, while persistent patients had a 29% reduction in overall fracture risk [6]. In a meta-analysis of six studies pooling over 171,000 patients, non-compliant patients had a 46% greater risk of experiencing fracture than their compliant counterparts, with the risk of vertebral fracture higher than hip or non-vertebral fracture [10]. Poor compliance has also been shown to affect BMD: among a population of nearly 250 women in a managed care plan, those receiving bisphosphonates or estrogen with MPR ≥66% had greater increases in BMD than those with MPR <66% [28]. Because the clinical benefit of osteoporosis therapies diminishes over time upon discontinuation, persistence and compliance with medications is critical in minimizing the risk of bone fractures [4].

This study provides an additional contribution to the literature with regard to long-term persistence and compliance among postmenopausal women receiving one of the currently available osteoporosis therapies. Payers, practitioners, and policy makers would benefit from an understanding of long-term persistence and its effect on treatment outcomes. Additional research is needed to understand factors associated with improved persistence and compliance.

### Limitations

This study has certain limitations. With regard to the osteoporosis therapies included in this study, it is important to note that because zoledronic acid is an annual therapy and the follow-up period in this study was limited to 24 months, this analysis may have underestimated persistence with zoledronic acid in patients whose gap periods overlapped with the end of the follow-up period. For risedronate daily, the sample size (*n* = 53) was too low to provide reliable comparisons.

In addition, because this study was conducted using administrative claims data from a working population of patients with employer-sponsored health insurance and Medicare patients with supplemental insurance paid for by their employers, this study population may be overall younger and healthier than patients with other types of health insurance or the uninsured. Moreover, claims data were generated for reimbursement purposes and may be subject to differences in billing and reimbursement practices. It should be noted that patients restarting their index osteoporosis therapy after a gap in therapy greater than the 14-month pre-index period may have been included in the study population and considered new to their index therapy. Results from this study may not be generalizable to osteoporosis patients without commercial or private Medicare supplemental coverage. Use of denosumab (Prolia®) before January 1, 2012, was captured by either a National Drug Code or a Healthcare Common Procedure Coding System (HCPCS) code for unclassified drugs (J3490 and J3590), where the HCPCS codes may have also been used to indicate other therapies (e.g., denosumab [Xgeva®] for bone problems secondary to cancer).

In this retrospective, observational study of women aged at least 50 years and initiating a new osteoporosis therapy, persistence and compliance at both 1 and 2 years were generally higher for patients receiving injectable rather than oral therapies. In particular, persistence and compliance for patients receiving an injectable therapy administered Q6M (i.e., denosumab) at 2 years was higher than for those receiving a daily injectable therapy (i.e., teriparatide) and oral therapies, especially oral bisphosphonates and raloxifene.

## References

[1] World Health Organization (2003) Prevention and management of osteoporosis: WHO Technical Report series no. 92. http://whqlibdoc.who.int/trs/who_trs_92pdf. Accessed 02 Feb 2016 15293701

[2] Cummings SR, Melton LJ (2002). Epidemiology and outcomes of osteoporotic fractures. Lancet.

[3] Scott LJ (2014). Denosumab: a review of its use in postmenopausal women with osteoporosis. Drugs Aging.

[4] National Osteoporosis Foundation (2014). 2014 Clinician’s guide to prevention and treatment of osteoporosis.

[5] Caro JJ, Ishak KJ, Huybrechts KF, Raggio G, Naujoks C (2004). The impact of compliance with osteoporosis therapy on fracture rates in actual practice. Osteoporos Int.

[6] Siris ES, Harris ST, Rosen CJ, Barr CE, Arvesen JN, Abbott TA, Silverman S (2006). Adherence to bisphosphonate therapy and fracture rates in osteoporotic women: relationship to vertebral and nonvertebral fractures from 2 US claims databases. Mayo Clin Proc.

[7] Cheng LI, Durden E, Limone B, Radbill L, Juneau PL, Spangler L, Mirza FM, Stolshek BS (2015). Persistance and compliance with osteroporosis therapies among women in a commercially insured population in the United States. J Manag Care Spec Pharm.

[8] Karlsson L, Lundkvist J, Psachoulia E, Intorcia M, Strom O (2015). Persistence with denosumab and persistence with oral bisphosphonates for the treatment of postmenopausal osteoporosis: a retrospective, observational study, and a meta-analysis. Osteoporos Int.

[9] Cotte FE, Fardellone P, Mercier F, Gaudin AF, Roux C (2010). Adherence to monthly and weekly oral bisphosphonates in women with osteoporosis. Osteoporos Int.

[10] Imaz I, Zegarra P, Gonzalez-Enriquez J, Rubio B, Alcazar R, Amate JM (2010). Poor bisphosphonate adherence for treatment of osteoporosis increases fracture risk: systematic review and meta-analysis. Osteoporos Int.

[11] Weycker D, Macarios D, Edelsberg J, Oster G (2006). Compliance with drug therapy for postmenopausal osteoporosis. Osteoporos Int.

[12] Confavreux CB, Canoui-Poitrine F, Schott AM, Ambrosi V, Tainturier V, Chapurlat RD (2012). Persistence at 1 year of oral antiosteoporotic drugs: a prospective study in a comprehensive health insurance database. Eur J Endocrinol.

[13] Devine J, Trice S, Finney Z, Yarger S, Nwokeji E, Linton A, Davies W (2012). A retrospective analysis of extended-interval dosing and the impact on bisphosphonate compliance in the US Military Health System. Osteoporos Int.

[14] Iolascon G, Gimigliano F, Orlando V, Capaldo A, Di Somma C, Menditto E (2013). Osteoporosis drugs in real-world clinical practice: an analysis of persistence. Aging Clin Exp Res.

[15] Li L, Roddam A, Gitlin M, Taylor A, Shepherd S, Shearer A, Jick S (2012). Persistence with osteoporosis medications among postmenopausal women in the UK General Practice Research Database. Menopause.

[16] Wade SW, Curtis JR, Yu J, White J, Stolshek BS, Merinar C, Balasubramanian A, Kallich JD, Adams JL, Viswanathan HN (2012). Medication adherence and fracture risk among patients on bisphosphonate therapy in a large United States health plan. Bone.

[17] Ziller V, Kostev K, Kyvernitakis I, Boeckhoff J, Hadji P (2012). Persistence and compliance of medications used in the treatment of osteoporosis--analysis using a large scale, representative, longitudinal German database. Int J Clin Pharmacol Ther.

[18] Freemantle N, Satram-Hoang S, Tang ET, Kaur P, Macarios D, Siddhanti S, Borenstein J, Kendler DL (2012). Final results of the DAPS (Denosumab Adherence Preference Satisfaction) study: a 24-month, randomized, crossover comparison with alendronate in postmenopausal women. Osteoporos Int.

[19] Kendler DL, McClung MR, Freemantle N, Lillestol M, Moffett AH, Borenstein J, Satram-Hoang S, Yang YC, Kaur P, Macarios D, Siddhanti S (2011). Adherence, preference, and satisfaction of postmenopausal women taking denosumab or alendronate. Osteoporos Int.

[20] Yu S, Burge RT, Foster SA, Gelwicks S, Meadows ES (2012). The impact of teriparatide adherence and persistence on fracture outcomes. Osteoporos Int.

[21] Kothawala P, Badamgarav E, Ryu S, Miller RM, Halbert RJ (2007). Systematic review and meta-analysis of real-world adherence to drug therapy for osteoporosis. Mayo Clin Proc.

[22] Silverman SL, Siris E, Kendler DL, Belazi D, Brown JP, Gold DT, Lewiecki EM, Papaioannou A, Simonelli C, Ferreira I, Balasubramanian A, Dakin P, Ho P, Siddhanti S, Stolshek B, Recknor C (2015). Persistence at 12 months with denosumab in postmenopausal women with osteoporosis: interim results from a prospective observational study. Osteoporos Int.

[23] Hadji P, Claus V, Ziller V, Intorcia M, Kostev K, Steinle T (2012). GRAND: the German retrospective cohort analysis on compliance and persistence and the associated risk of fractures in osteoporotic women treated with oral bisphosphonates. Osteoporos Int.

[24] Landfeldt E, Strom O, Robbins S, Borgstrom F (2012). Adherence to treatment of primary osteoporosis and its association to fractures—the Swedish Adherence Register Analysis (SARA). Osteoporos Int.

[25] Soong YK, Tsai KS, Huang HY, Yang RS, Chen JF, Wu PC, Huang KE (2013). Risk of refracture associated with compliance and persistence with bisphosphonate therapy in Taiwan. Osteoporos Int.

[26] Seeman E, Compston J, Adachi J, Brandi ML, Cooper C, Dawson-Hughes B, Jonsson B, Pols H, Cramer JA (2007). Non-compliance: the Achilles’ heel of anti-fracture efficacy. Osteoporos Int.

[27] Siris ES, Selby PL, Saag KG, Borgstrom F, Herings RM, Silverman SL (2009). Impact of osteoporosis treatment adherence on fracture rates in North America and Europe. Am J Med.

[28] Yood RA, Andrade SE, Mazor KM, Fouayzi H, Chan W, Kahler K (2010). Bone density consequences of initiation and compliance with therapy for osteoporosis. Arthritis Care Res (Hoboken).

